# Piperlongumine-Induced Phosphatidylserine Translocation in the Erythrocyte Membrane

**DOI:** 10.3390/toxins6102975

**Published:** 2014-10-14

**Authors:** Rosi Bissinger, Abaid Malik, Jamshed Warsi, Kashif Jilani, Florian Lang

**Affiliations:** 1 Department of Physiology, Eberhard-Karls-University of Tuebingen, Gmelinstr. 5, 72076 Tuebingen, Germany; E-Mails: ro.bissinger@gmx.de (R.B.); malik.abaid@googlemail.com (A.M.); jamshedwarsi@yahoo.com (J.W.); kashif_cbc@yahoo.com (K.J.); 2 Department of Biochemistry, University of Agriculture, 38040 Faisalabad, Pakistan

**Keywords:** phosphatidylserine, piperlongumine, calcium, ceramide, cell volume, eryptosis

## Abstract

*Background*: Piperlongumine, a component of Piper longum fruit, is considered as a treatment for malignancy. It is effective by inducing apoptosis. Mechanisms involved in the apoptotic action of piperlongumine include oxidative stress and activation of p38 kinase. In analogy to apoptosis of nucleated cells, erythrocytes may undergo eryptosis, the suicidal death of erythrocytes characterized by cell shrinkage and cell membrane scrambling with phosphatidylserine-exposure at the erythrocyte surface. Signaling involved in eryptosis include increase of cytosolic Ca^2+^-activity ([Ca^2+^]*_i_*), formation of ceramide, oxidative stress and activation of p38 kinase. *Methods*: Cell volume was estimated from forward scatter, phosphatidylserine-exposure from annexin V binding, [Ca^2+^]*_i_* from Fluo3 fluorescence, reactive oxygen species from 2',7'-dichlorodihydrofluorescein-diacetate fluorescence, and ceramide abundance from binding of fluorescent antibodies in flow cytometry. *Results*: A 48 h exposure to piperlongumine (30 µM) was followed by significant decrease of forward scatter and increase of annexin-V-binding. Piperlongumine did not significantly modify [Ca^2+^]*_i_* and the effect was not dependent on presence of extracellular Ca^2+^. Piperlongumine significantly increased ROS formation and ceramide abundance. *Conclusions*: Piperlongumine triggers cell membrane scrambling, an effect independent from entry of extracellular Ca^2+^ but at least partially due to ROS and ceramide formation.

## 1. Introduction

Piper longum fruit has been and is used for the treatment of multiple disorders including chronic bronchitis, asthma, constipation, gonorrhea, paralysis of the tongue, diarrhea, cholera, chronic malaria, viral hepatitis, respiratory infections, stomachache, bronchitis, diseases of the spleen, cough, and tumors [1]. Piperlongumine (piplartine), an amide alkaloid component of Piper longum fruit [1], has particularly strong cytotoxic activity both, *in vitro* and *in vivo* [2,3,4,5] and enhances the sensitivity of tumor cells to cytostatic treatment [6]. Piperlongumine is at least partially effective by inducing apoptosis of tumor cells [2,7,8,9,10,11,12,13,14]. Mechanisms involved in the triggering of apoptosis by piperlongumine include induction of oxidative stress [6,7,9,12,15,16,17]. Piperlongumine may bind in tumor cells to the active sites of several key cellular antioxidants including glutathione S transferase and carbonyl reductase 1 [18]. Beyond that, piperlongumine inhibits PI3K/Akt/mTOR signalling leading to down-regulation of the NF-kB pathway, altered gene expression and activation of the mitochondrial apoptotic pathway [3,4,8,14,19,20]. Piperlongumine further activates p38 kinase [15,17], inhibits proteasomal protein degradation [10], and suppresses the transcription factor signal transducer and activator of transcription (STAT) 3 thus modulating expression of multiple Stat3-regulated genes [2].

Similar to apoptosis of nucleated cells, erythrocytes could enter eryptosis, the suicidal erythrocyte death characterized by cell shrinkage and cell membrane scrambling with phosphatidylserine exposure to the erythrocyte surface [21]. Eryptosis could be triggered by increase of cytosolic Ca^2+^ concentration ([Ca^2+^]*_i_*) [22], ceramide formation [21], caspase activation [23,24,25,26,27], lack of AMP activated kinase AMPK [28], stimulation of casein kinase 1α [29,30], lack of cGMP-dependent protein kinase [31], stimulation of Janus-activated kinase JAK3 [32], stimulation of protein kinase C [33], stimulation of p38 kinase [34], inhibition of PAK2 kinase [35] as well as inhibition of sorafenib [36] and sunitinib [37] sensitive kinases.

To the best of our knowledge, an effect of piperlongumine on suicidal erythrocyte death has never been reported before. The purpose of the present study was to explore whether piperlongumine triggers eryptosis, *i.e.*, the suicidal death of cells devoid of mitochondria and nuclei. Moreover, the study explored the involvement of [Ca^2+^]_i_, ROS formation and ceramide abundance.

## 2. Results and Discussion

**Figure 1:**
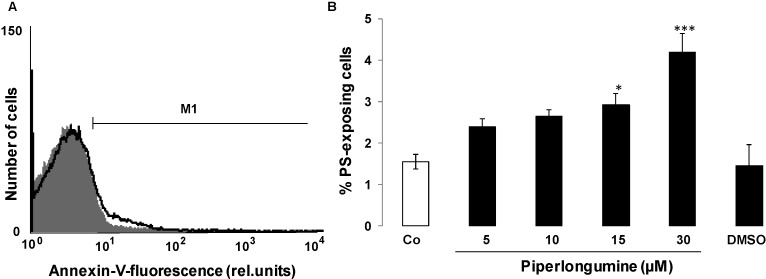
Effect of piperlongumine on phosphatidylserine exposure (**A**) Original histogram of annexin V binding of erythrocytes following exposure for 48 h to Ringer solution without (grey shadow) and with (black line) presence of 30 µM piperlongumine; (**B**) Arithmetic means ± SEM (*n* = 15) of erythrocyte annexin-V-binding following incubation for 48 h to Ringer solution without (white bar) or with (black bars) presence of piperlongumine (5–30 µM) or, for comparison, DMSO (0.1%) alone. * (*p* < 0.05), *** (*p* < 0.001) indicate significant differences from the absence of piperlongumine (ANOVA).

The present study explored the putative effect of piperlongumine on eryptosis, the suicidal erythrocyte death. Hallmarks of eryptosis include breakdown of phosphatidylserine asymmetry of the erythrocyte cell membrane with subsequent translocation of phosphatidylserine to the cell surface. Phosphatidylserine exposing erythrocytes were identified by annexin-V-binding in flow cytometry. As illustrated in Figure 1, a 48 h exposure to piperlongumine increased the percentage of annexin-V-binding erythrocytes, an effect reaching statistical significance at 15 µM piperlongumine concentration.

**Figure 2:**
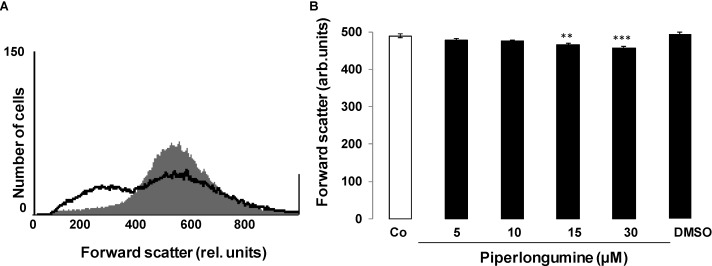
Effect of piperlongumine on erythrocyte forward scatter (**A**) Original histogram of forward scatter of erythrocytes following exposure for 48 h to Ringer solution without (grey shadow) and with (black line) presence of 30 µM piperlongumine; (**B**) Arithmetic means ± SEM (*n* = 15) of the normalized erythrocyte forward scatter (FSC) following incubation for 48 h to Ringer solution without (white bar) or with (black bars) piperlongumine (5–30 µM) or, for comparison, DMSO (0.1%) alone. ** (*p* < 0.01), *** (*p* < 0.001) indicate significant differences from the absence of piperlongumine (ANOVA).

A further hallmark of eryptosis is cell shrinkage. Cell volume was thus estimated from forward scatter in flow cytometry. As shown in Figure 2, a 48 h exposure to piperlongumine slightly decreased average forward scatter, an effect reaching statistical significance at 15 µM piperlongumine concentration. Closer inspection of the histogram reveals that the forward scatter decreases substantially in a minor portion of erythrocytes, whereas the forward scatter of most erythrocytes does not appreciably change. Thus, the average forward scatter underestimates the effect of piperlongumine on those erythrocytes which undergo eryptosis.

**Figure 3:**
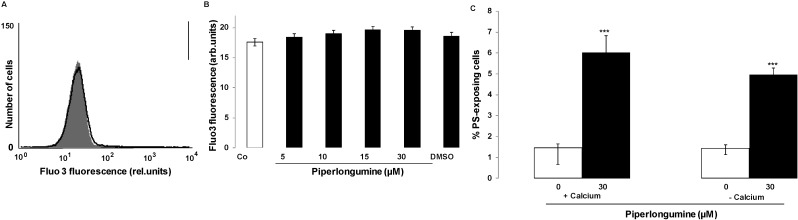
Effect of piperlongumine on erythrocyte cytosolic Ca^2+^ concentration (**A**) Original histogram of Fluo3 fluorescence in erythrocytes following exposure for 48 h to Ringer solution without (grey shadow) and with (black line) presence of 30 µM piperlongumine; (**B**) Arithmetic means ± SEM (*n* = 15) of the Fluo3 fluorescence (arbitrary units) in erythrocytes exposed for 48 h to Ringer solution without (white bar) or with (black bars) piperlongumine (5–30 µM) or, for comparison, DMSO (0.1%) alone; (**C**) Arithmetic means ± SEM (*n* = 5) of the percentage of annexin-V-binding erythrocytes after a 48 h treatment with Ringer solution without (white bars) or with (black bars) 30 µM piperlongumine in the presence (left bars, +Calcium) and absence (right bars, −Calcium) of calcium. *** (*p* < 0.001) indicates significant difference from the respective values in the absence of piperlongumine (ANOVA).

Both, cell membrane scrambling and cell shrinkage could have resulted from increase of cytosolic Ca^2+^ activity ([Ca^2+^]*_i_*). In order to estimate [Ca^2+^]*_i_*, erythrocytes were loaded with Fluo3-AM and Fluo3 fluorescence determined by flow cytometry. As illustrated in Figure 3A,B, a 48 h exposure of human erythrocytes to piperlongumine tended to slightly increase Fluo3 fluorescence, an effect, however, not reaching statistical significance even at the highest concentration (30 µM) employed.

Additional experiments explored, whether the piperlongumine induced cell membrane scrambling required entry of extracellular Ca^2+^. To this end, erythrocytes were exposed for 48 h to 30 µM piperlongumine in the presence or nominal absence of extracellular Ca^2+^. As shown in Figure 3C, the effect of piperlongumine on annexin-V-binding tended to be lower in the nominal absence than in the presence of Ca^2+^, an effect, however, not reaching statistical significance. In the nominal absence of extracellular Ca^2+^, piperlongumine still significantly increased the percentage of annexin V binding erythrocytes. Thus, the effect of piperlongumine on cell membrane scrambling did not critically depend on Ca^2+^ entry.

In order to determine the effect of piperlongumine exposure on hemolysis, the percentage of hemolysed erythrocytes was quantified from hemoglobin concentration in the supernatant. According to the hemoglobin concentration, a 48 h incubation with 0, 5, 10, 15 and 30 µM piperlongumine resulted in hemolysis of 2.4% ± 0.4%, 2.6% ± 0.3%, 2.5% ± 0.5%, 2.5% ± 0.2% and 4.6% ± 1.5% (*n* = 4), respectively.

**Figure 4:**
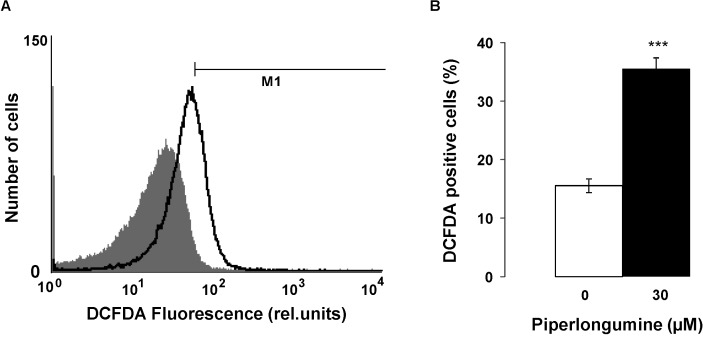
Effect of piperlongumine on reactive oxygen species (**A**) Original histogram of 2',7'-dichlorodihydrofluorescein diacetate (DCFDA) fluorescence in erythrocytes following exposure for 48 h to Ringer solution without (grey shadow) and with (black line) presence of 30 µM piperlongumine; (**B**) Arithmetic means ± SEM (*n* = 5) of erythrocyte DCFDA fluorescence following incubation for 48 h to Ringer solution without (white bar) or with (black bar) presence of piperlongumine (30 µM). *** (*p* < 0.001) indicates significant difference from the absence of piperlongumine (ANOVA).

In search for further mechanisms involved in the effect of piperlongumine on eryptosis, reactive oxygen species (ROS) were determined utilizing 2',7'-dichlorodihydrofluorescein diacetate (DCFDA). As shown in Figure 4, a 48 h exposure to 30 µM piperlongumine markedly and significantly increased the DCFDA fluorescence, a finding pointing to induction of oxidative stress. The percentage of oxidized erythrocytes was higher than the percentage of erythrocytes with translocated phosphatidylserine. Accordingly, strong ROS formation was apparently required to stimulate cell membrane scrambling.

**Figure 5:**
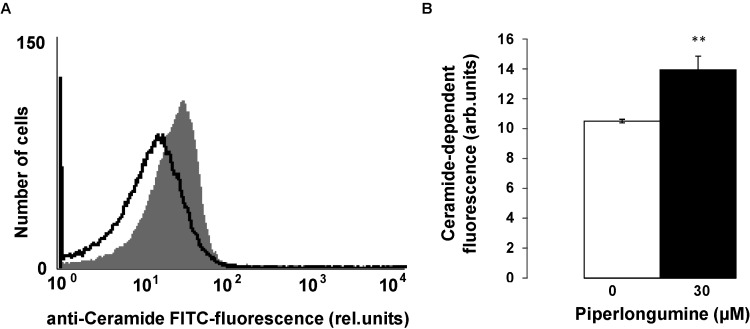
Effect of piperlongumine on ceramide abundance (**A**) Original histogram of anti-ceramide fluorescein isothiocyanate (FITC) fluorescence in erythrocytes after exposure for 48 h to Ringer solution without (grey shadow) and with (black line) presence of 30 µM piperlongumine; (**B**) Arithmetic means ± SEM (*n* = 9) of ceramide abundance at the erythrocyte surface following incubation for 48 h to Ringer solution without (white bar) or with (black bar) presence of piperlongumine (30 µM). ** (*p* < 0.01) indicates significant difference from the absence of piperlongumine (ANOVA).

As cell membrane scrambling could be further elicited even at constant [Ca^2+^]_i_ by ceramide, additional experiments explored, whether piperlongumine increases ceramide formation. The abundance of ceramide at the erythrocyte surface was visualized with an anti-ceramide antibody. As shown in Figure 5, a 48 h exposure to 30 µM piperlongumine significantly increased the ceramide abundance at the erythrocyte surface.

The present study reveals that exposure of human erythrocytes to piperlongumine is followed by eryptosis, the suicidal erythrocyte death characterized by cell shrinkage and cell membrane scrambling with phosphatidylserine translocation to the erythrocyte surface. The piperlongumine concentrations required for the triggering of eryptosis are within the range of piperlongumine concentrations employed to elicit apoptosis of tumor cells [20].

Piperlongumine did not significantly increase cytosolic Ca^2+^ activity ([Ca^2+^]*_i_*), a major trigger of eryptosis [21]. Instead, piperlongumine increased the abundance of ceramide and induced oxidative stress, both well known triggers of eryptosis [21]. In tumor cells, the apoptotic effect of piperlongumine similarly involves oxidative stress [6,7,9,12,15,16,17]. To the best of our knowledge, an effect of piperlongumine on ceramide formation has never been shown before. It would thus be interesting to learn whether piperlongumine triggers ceramide formation as well in nucleated cells.

The phosphatidylserine exposing erythrocytes attach to respective receptors of phagocytes with subsequent engulfment and degradation of the defective erythrocytes [21]. Thus, eryptotic phosphatidylserine exposing erythrocytes are rapidly cleared from circulating blood [21]. The eryptotic cell shrinkage [22] counteracts swelling of the defective cells, which would otherwise lead to rupture of the cell membrane with release of cellular hemoglobin. Released hemoglobin may be filtered in renal glomeruli, subsequently precipitate in the acidic lumen of renal tubules and thus occlude the tubules [38].

Excessive eryptosis may become pathophysiologically relevant. The clearance of phosphatidylserine exposing erythrocytes from circulating blood may lead to anemia as soon as the loss of eryptotic erythrocytes fails to be outweighed by similar formation of new erythrocytes [21]. Treatment of erythrocytes with 15 or 30 µM piperlongumine approximately doubles the percentage of phosphatidylserine exposing erythrocytes. In the absence of compensatory increase of erythropoiesis, the effect would halve the number of erythrocytes in circulating blood and thus lead to severe anemia. Phosphatidylserine exposing erythrocytes may further adhere to endothelial receptors [39]. The attachment of eryptotic erythrocytes to the vascular wall compromises microcirculation [39,40,41,42,43,44]. Moreover, phosphatidylserine exposing erythrocytes stimulate blood clotting and thus thrombosis [40,45,46].

The sensitivity of erythrocytes to piperlongumine may be augmented by additional exposure to further eryptosis triggering xenobiotics [21,37,47,48,49,50,51,52,53,54,55,56,57,58,59,60,61,62,63,64,65,66,67,68,69,70,71,72,73,74,75,76,77,78,79,80] or in patients suffering from disorders facilitating eryptosis [21], such as diabetes [27,81,82], renal insufficiency [83], hemolytic uremic syndrome [84], sepsis [85], malaria [86], sickle cell disease [86], Wilson’s disease [87], iron deficiency [88], malignancy [89], phosphate depletion [90], and metabolic syndrome [65].

## 3. Experimental Section

### 3.1. Erythrocytes, Solutions and Chemicals

Fresh Li-Heparin-anticoagulated blood samples were kindly provided by the blood bank of the University of Tübingen. The study is approved by the ethics committee of the University of Tübingen (184/2003V). The blood was centrifuged at 120 rcf for 20 min at 23 °C and the platelets and leukocytes-containing supernatant were disposed. Erythrocytes were washed in Ringer solution containing (in mM) 125 NaCl, 5 KCl, 1 MgSO_4_, 32 *N*-2-hydroxyethylpiperazine-*N*-2-ethanesulfonic acid (HEPES, pH 7.4), 5 glucose, and 1 CaCl_2_. For the experiments, erythrocytes were incubated *in vitro* at a hematocrit of 0.4% at 37 °C for 48 h. Where indicated, erythrocytes were exposed to piperlongumine (Sigma-Aldrich, Hamburg, Germany) at the indicated concentrations. In Ca^2+^-free Ringer solution, 1 mM CaCl_2_ was substituted by 1 mM glycol-bis(2-aminoethylether)-*N*,*N*,*N*',*N*'-tetraacetic acid (EGTA).

### 3.2. FACS Analysis of Annexin-V-Binding and Forward Scatter

After incubation under the respective experimental condition, 50 µL cell suspension was washed in Ringer solution containing 5 mM CaCl_2_ and then stained with Annexin-V-FITC (1:200 dilution; ImmunoTools, Friesoythe, Germany) in this solution at 37 °C for 20 min under protection from light. In the following, the forward scatter (FSC) of the cells was determined, and annexin-V fluorescence intensity was measured with an excitation wavelength of 488 nm and an emission wavelength of 530 nm on a FACS Calibur (BD, Heidelberg, Germany).

### 3.3. Measurement of Intracellular Ca^2+^

After incubation, erythrocytes were washed in Ringer solution and then loaded with Fluo-3/AM (Biotium, Hayward, CA, USA) in Ringer solution containing 5 mM CaCl_2_ and 5 µM Fluo-3/AM. The cells were incubated at 37 °C for 30 min and washed twice in Ringer solution containing 5 mM CaCl_2_. The Fluo-3/AM-loaded erythrocytes were resuspended in 200 µL Ringer. Then, Ca^2+^-dependent fluorescence intensity was measured with an excitation wavelength of 488 nm and an emission wavelength of 530 nm on a FACS Calibur. The adequacy of Fluo-3 fluorescence as a tool to estimate cytosolic Ca^2+^ activity has been tested in several previous studies [29,91,92,93,94].

### 3.4. Determination of ROS Production

ROS production was determined utilizing 2',7'-dichlorodihydrofluorescein diacetate (DCFDA) [95]. Briefly, the cells were suspended in FACS buffer and the fluorescence was analysed with flow cytometry (FACS-calibur from Becton Dickinson; Heidelberg, Germany). DCFDA fluorescence intensity was measured in FL-1 with an excitation wavelength of 488 nm and an emission wavelength of 530 nm.

### 3.5. Determination of Ceramide Formation

For the determination of ceramide, a monoclonal antibody-based assay was used. After incubation, cells were stained for 1 h at 37 °C with 1 µg/mL anti ceramide antibody (clone MID 15B4, Alexis, Grünberg, Germany) in PBS containing 0.1% bovine serum albumin (BSA) at a dilution of 1:10. The samples were washed twice with PBS-BSA. Subsequently, the cells were stained for 30 min with polyclonal fluorescein isothiocyanate (FITC) conjugated goat anti-mouse IgG and IgM specific antibody (Pharmingen, Hamburg, Germany) diluted 1:50 in PBS-BSA. Unbound secondary antibody was removed by repeated washing with PBS-BSA. The samples were then analyzed by flow cytometric analysis with an excitation wavelength of 488 nm and an emission wavelength of 530 nm.

### 3.6. Measurement of Hemolysis

For the determination of hemolysis, the samples were centrifuged (3 min at 1600 RPM, room temperature) after incubation, and the supernatants were harvested. As a measure of hemolysis, the hemoglobin (Hb) concentration of the supernatant was determined photometrically at 405 nm. The absorption of the supernatant of erythrocytes lysed in distilled water was defined as 100% hemolysis.

### 3.7. Statistics

Data are expressed as arithmetic means ± SEM. As indicated in the figure legends, statistical analysis was made using ANOVA with Tukey’s test as post-test and *t* test as appropriate. The number of different erythrocyte specimens studied is given as n. Since different erythrocyte specimens used in distinct experiments are differently susceptible to triggers of eryptosis, the same erythrocyte specimens have been used for control and experimental conditions.

## 4. Conclusions

In conclusion, the present paper unequivocally and significantly demonstrates that piperlongumine stimulates eryptosis with erythrocyte shrinkage and cell membrane scrambling. Thus, piperlongumine is able to trigger suicidal death in cells lacking mitochondria and nuclei. The cellular mechanisms involved include oxidative stress and ceramide formation. To the best of our knowledge, an effect of piperlongumine on suicidal erythrocyte death or of ceramide formation in any cell type has never been reported before.
